# Investigating the effects of fire on pollinator‐dependent distyly polymorphism

**DOI:** 10.1111/plb.70062

**Published:** 2025-07-04

**Authors:** R. Trevizan, C. Mendes‐Rodrigues, P. E. Oliveira, P. K. Maruyama, F. W. Amorim, J. C. F. Cardoso

**Affiliations:** ^1^ Programa de Pós‐Graduação em Biologia Vegetal Universidade Estadual de Campinas Campinas Brazil; ^2^ Programa de Pós‐Graduação em Ecologia, Conservação e Manejo da Fauna Silvestre Universidade Federal de Minas Gerais Belo Horizonte Brazil; ^3^ Centro de Síntese Ecológica e Conservação, Departamento de Genética, Ecologia e Evolução, ICB Universidade Federal de Minas Gerais Belo Horizonte Brazil; ^4^ Instituto de Biologia Universidade Federal de Uberlândia, Campus Umuarama Uberlândia Minas Gerais Brazil; ^5^ Faculdade de Medicina Universidade Federal de Uberlândia Uberlândia Brazil; ^6^ Laboratório de Ecologia da Polinização e Interações—LEPI, Departamento de Biodiversidade e Bioestatística, Instituto de Biociências Universidade Estadual Paulista ‘Júlio de Mesquita Filho’ (UNESP) São Paulo Brazil

**Keywords:** Brazilian savanna, Cerrado, distyly, fire‐prone ecosystems, morphological traits, reciprocity

## Abstract

- Fire influences plant traits in several ways, but its effects on flower polymorphisms are unknown. Distyly, a floral polymorphism with long‐styled (L‐styled) and short‐styled (S‐styled) morphs exhibiting reciprocal herkogamy and a self‐incompatibility system, depends on biotic pollination for intermorph pollination and reproduction. We investigated the effects of fire on the functioning of distyly, assessing morphology, floral reward, between‐morph reciprocity, and reproductive success.
- We studied a population of the distylous hummingbird‐pollinated *Palicourea rigida* (Rubiaceae) in the Brazilian savanna by comparing individuals from a fire‐affected area with those from a contiguous unaffected area.
- Fire affected some floral traits, reducing the number of inflorescences (9.4%), corollas (4.7%), anthers (5.9%), and L‐styled stigmas (33.5%). However, it did not affect plant height, number of buds and inflorescences, or nectar traits. Fire did not affect between‐morph reciprocity. Hence, plants affected or not affected by fire presented similar reciprocity measures, translating into similar pollen deposition and fruit set of both morphs. Fire also had a morph‐specific positive effect on the S‐morph fruit set (35.31% increase).
- Fire can induce morphological changes in distylous species. However, fire does not influence most pollinator attraction and reward traits. The consistency of reproductive heights enables pollen flow within the fire‐affected area and across the mosaic of different fire histories. The high resprouting ability linked to the rapid restoration of reproductive capacity allows distyly functioning through efficient intermorph pollen transfer. Such plant resilience may be important for maintaining the polymorphism and the associated pollinators under increasingly frequent anthropogenic fires.

## Graphical abstract

While fire causes some morphological changes in pollinator‐dependent distylous flowers, most traits related to attraction, reward, and stigma‐anther reciprocity remain unaffected, ensuring intermorph pollen transfer and maintaining the floral polymorphism.

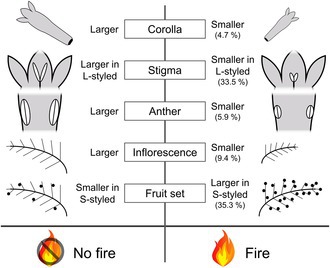

## INTRODUCTION

Fire is a fundamental natural disturbance in fire‐prone ecosystems, shaping the diversity and dynamics of communities (Kelly & Brotons 2017; Carbone *et al*. 2019; Kobziar *et al*. 2024). Many plant species in these ecosystems have evolved adaptive strategies mediated by the selective pressure of fire. These include resistance and protection structures, such as underground organs, thick barks, sparse branching and leaves, and leaves that grow on shoot tips (Simon *et al*. 2009; Pausas 2015). There are even plants dependent on fire, with some species breaking seed dormancy and germinating when triggered by fire or smoke (Pausas 2019; Wigley *et al*. 2021). Another common response includes flowering after resprouting, with some species dependent on or stimulated by fire (Fidelis *et al*. 2019; Fidelis & Zirondi 2021; Wigley *et al*. 2021; Zirondi *et al*. 2021). Finally, the spatial and temporal variability of fire in the landscape can influence flowering and fruiting patterns among different species, enhancing species diversity and coexistence within the community (Ferreira *et al*. 2023).

Although fire response strategies are well understood in plants, knowledge of fire effects in ecological interactions remains limited (Driscoll *et al*. 2010; Pausas 2019; Ballarin *et al*. 2024; Carbone *et al*. 2025). This is the case for pollination, an important interaction in ecosystems that promotes the reproduction of plants through the transport of pollen and the maintenance of pollinator populations via the exploitation of floral resources (Ollerton 2021). The effects of fire vary across populations of both pollinators and plants. While some pollinator groups disappear, others can increase in diversity and abundance, which can be explained by factors such as flight capacity, nesting, or feeding habits (Pausas & Parr 2018; Burkle *et al*. 2019; Carbone *et al*. 2025). Regarding plants, positive fire responses include a higher richness and abundance of flowers, improved visitation rate, and enhanced reproductive success (Fidelis & Blanco 2014; Fidelis & Zirondi 2021; Ferreira *et al*. 2024; Carbone *et al*. 2025; Tosatto *et al*. 2025). However, the effects of fire can be idiosyncratic and negative for plant populations by reducing pollination or even resulting in local extinctions (Fidelis & Zirondi 2021).

**Fig. 1:**
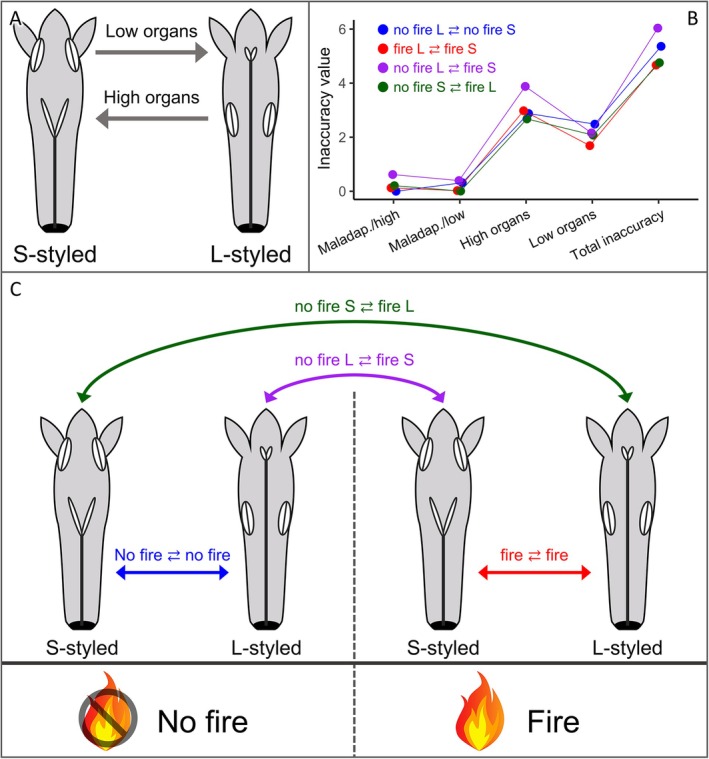
(A) Representation of distyly polymorphism, showing L‐styled morph, S‐styled morph, and the reciprocal hercogamy between reproductive structures (high organs and low organs). Arrows indicate the direction of pollen flow. (B) Inaccuracy values for all possible legitimate pollen transfer combinations between plants from fire and no‐fire areas. Maladaptive bias is abbreviated as ‘Maladap’ for both high and low organs. (C) Schematic representation of these combinations.

Self‐incompatible plants are obligatorily dependent on pollination, producing viable seeds exclusively through outcrossing. In this case, a fire event can have a positive outcome if it enhances pollination (Ne'eman *et al*. 2000). Conversely, it can have negative effects if it diminishes pollination services, thereby reducing fruit or seed production (Ne'eman *et al*. 2000). However, the effects of fire on the pollination of plants with self‐incompatible systems are still underexplored, especially concerning specialized floral polymorphisms. Distyly is the most common floral polymorphism, where individual plants have two co‐dependent floral morphs within the same population: a short‐styled morph (S‐styled), with stigmas below the anthers, and a long‐styled morph (L‐styled), with stigmas above the anthers (Fig. 1A; Darwin 1877; Barrett 2019). The morphs are characterized by the correspondent arrangement between anthers and stigmas, that is, reciprocal herkogamy, being generally accompanied by a heteromorphic self‐incompatibility system, making the animal‐mediated intermorph pollination mandatory for sexual reproduction and maintenance of populations (Keller *et al*. 2014; Trevizan *et al*. 2021b).

Fire can affect traits important in pollination, such as number of flowers per inflorescence and flower size (Burkle *et al*. 2019). Since reciprocal herkogamy is essential to intermorph pollination in distylous plants, height mismatches in anthers and stigmas between morphs can lead to insufficient legitimate pollination through pollen wastage and increase the chances of illegitimate (intramorph) pollination through pollen discounting (Darwin 1877; Lloyd & Webb 1992; Keller *et al*. 2014; Armbruster *et al*. 2017). Therefore, the arrangement of flower morphological traits is important for the functioning of distyly. In a post‐fire scenario, plants can have their flower morphology affected, for example, by growing larger flowers due to the increased availability of nutrients from ash (Burkle *et al*. 2019; Carbone *et al*. 2025), or by increasing smaller flower numbers due to limitations or constraints on resprouting. Also, morphs can have their floral traits affected differentially according to external factors, such as herbivory (Leege & Wolfe 2002). Thus, fire may affect the pollen flow of distylous plants, especially considering that fire‐prone ecosystems have a pattern of burning in mosaics, creating burned and unburned patches in the landscape (Kauffman *et al*. 1994; Ponisio *et al*. 2016).

The Brazilian Cerrado is a savanna ecosystem that has experienced fire regimes for thousands of years (Miranda *et al*. 2002; Simon *et al*. 2009). Fire has been considered an essential factor in regulating plant biomass, maintaining the typical savanna structure and ecosystem functioning in the Cerrado (Bond & Keeley 2005; Durigan & Ratter 2016; Pilon *et al*. 2021; Rodrigues & Fidelis 2022). Most Cerrado plants depend on the biotic pollination service to produce seeds and have several strategies to promote cross‐pollination (Oliveira & Gibbs 2000; Barbosa & Sazima 2008). Distyly is by far the most frequently reported floral polymorphism in the Cerrado (Cardoso *et al*. 2022), making it important to understand how fire affects this widespread system that depends obligatorily on specific floral morphology arrangements and pollinators.

To fill knowledge gaps regarding the effects of fire on self‐incompatible and floral polymorphic plants, we used the hummingbird‐pollinated *Palicourea rigida* (Rubiaceae) as a model species, which is abundant in Cerrado areas and represents one of the world's most studied distylous plants. To thoroughly study the effects of fire on ecological interactions, it is necessary to have appropriate controls, such as simultaneous comparisons of burned and unburned areas (Ballarin *et al*. 2024). Thus, we used a population where some individuals were fire‐affected while others remained untouched and intact, constituting two areas separated by ca. 10 m. Based on this design, we aimed to investigate the effects of fire, examining whether this disturbance has a positive, negative, or neutral effect on functioning of distyly. We investigated traits of morphology, floral reward, between‐morph reciprocity, and estimated reproductive success through pollen deposition and fruit formation. Since fire has been present in the Cerrado for millennia, plants have evolved several adaptations to survive fire regimes (Simon *et al*. 2009). Based on this, our research hypothesis is that fire does not strongly influence plant and floral traits, especially reproductive whorls, retaining between‐morph reciprocity within and across areas. This will translate into the maintenance of intermorph pollen flow, sexual reproduction and, consequently the functioning of distyly.

## MATERIAL AND METHODS

### Study area

We conducted the study at Caldas Novas State Park (PESCAN, 17°47′S; 48°40′W), in Caldas Novas city, southeast region of Goiás, Brazil. The reserve has an area of 12.3 ha, with elevation varying from 758 to 1047 m a.s.l. (Cardoso *et al*. 2022). The region has an AW climate type according to then Köppen classification, with a hot and rainy summer from October to March and a hot and dry winter from April to September (Alvares *et al*. 2013).

The study was conducted on a plateau at ~1047 m a.s.l. in a Cerrado *sensu strictu*, characterized by sparsely distributed tortuous shrubs growing among herbaceous and grassy plants (Cardoso *et al*. 2022). The area is dominated by a continuous layer of fast‐flammable grasses (Pausas *et al*. 2017). On 12 August 2006, the region had a fire of anthropic origin (Lopes *et al*. 2009). This consumed the area on one side of a narrow road where the soil is exposed, which functioned as a barrier preventing the spread of fire to the other side, creating an unburned area that remained unburned for ca. 10 years before data collection (Lopes *et al*. 2009; Altomare *et al*. 2021). This created two nearby areas comprising the same plant population separated by ca. 10 m, with similar biotic and abiotic conditions, differing only in the influence of fire (see Altomare *et al*. 2021). Field sampling was conducted in October 2006.

### Study species

*Palicourea rigida* Kunth is a treelet widely distributed in the Cerrado (Bridgewater *et al*. 2004), with peak flowering between October and December (Machado *et al*. 2010; Cardoso *et al*. 2022). After the fast‐passing fire in the herbaceous layer, *P. rigida* individuals usually show crown or basal resprouting (Medeiros & Miranda 2008). Due to their low flammability characteristics, including high water content, and sturdy bark that protects the buds from fire (Souza & Vale 2019), the plants did not experience top‐kill when the fire passed. Instead, the aerial part was partially burned, and shortly afterward, sprouted and flowered.

The *P. rigida* population is characterized by typically ornithophilous flowers, with S‐styled flowers measuring, on average, 16.15 mm, and L‐styled flowers measuring 16.85 mm. These flowers are tubular, brightly coloured, and odourless, attracting several hummingbird species (Machado *et al*. 2010; Justino *et al*. 2012; Maruyama *et al*. 2016; Trevizan *et al*. 2024). Flowers last 1 day, opening at dawn and wilting at the end of the day (Machado *et al*. 2010). The species is self‐incompatible, requiring intermorph pollination to set fruit, and the studied population is typically distylous and isoplethic, with balanced frequencies of floral morphs (L‐ and S‐styled), and exhibits well‐defined reproductive organ heights and reciprocity (Machado *et al*. 2010; Cardoso *et al*. 2022; Trevizan *et al*. 2024).

### Individual traits

We sampled 69 individuals from the no‐fire and fire‐affected areas to evaluate individual traits, sampling ca. 17 individuals per morph per area (Table S1). We recorded plant height and stem diameter, with plant height measured from the base of the individual to the top of the canopy, and the stem diameter measured at 30 cm above the ground. Additionally, we counted the number of inflorescences for each individual, as a proxy for floral display (Table S1). We also collected inflorescence length and number of buds from 89 inflorescences/individuals with ca. 22 inflorescences per morph per area (Table S1). Finally, we counted the total number of fruits on 142 inflorescences from ca. 36 inflorescences, and ca. 11 individuals per morph per area (Table S1).

### Floral traits and between‐morph reciprocity

To evaluate floral traits, we collected 321 flowers from 10 individuals and ca. 80 flowers per morph per area (Table S1). We measured corolla length (from corolla base to petal opening), corolla diameter (corolla opening), anther length, stigma length, stigma height (from base of the corolla to apex of the stigma), and anther height (from base of the corolla to apex of the anthers). The floral trait measurements were taken using a digital calliper (0.01 mm readability).

We used the height of reproductive whorls to measure reciprocity between morphs based on adaptive accuracy theory (*sensu* Armbruster *et al*. 2017). Accordingly, the greater the inaccuracy values, the lower the reciprocity between anthers and stigmas of opposite morphs (Armbruster *et al*. 2017). Inaccuracy was calculated based on means and variances of anther and stigma heights of L‐ and S‐styled morphs. Based on our design, which includes areas in two conditions, we calculated inaccuracy for all four possible between‐morph (legitimate) combinations, including: within the no‐fire area (no‐fire L ⇄ no‐fire S), within the fire‐affected area (fire L ⇄ fire S), between the S‐morph of the no‐fire area and the L‐morph of the fire area (no‐fire S ⇄ fire L), and between the L‐morph of the no‐fire area and the S‐morph of the fire area (no‐fire L ⇄ fire S) (see Fig. 1C), as follows:

Inaccuracy_no‐fire L ⇄ no‐fire S_:

$${\text{Inaccuracy}}_{\text{High}}={\left({\bar{A}}_{\mathrm{no}-\text{fire}}-{\bar{S}}_{\mathrm{no}-\text{fire}}\right)}^{2}+V_{A}+V_{S}$$

$${\text{Inaccuracy}}_{\mathrm{Low}}={\left({\bar{a}}_{\mathrm{no}-\text{fire}}-{\bar{s}}_{\mathrm{no}-\text{fire}}\right)}^{2}+V_{a}+V_{s}$$

Inaccuracy_fire L ⇄ fire S_:

$${\text{Inaccuracy}}_{\text{High}}={\left({\bar{A}}_{\text{fire}}-{\bar{S}}_{i}\right)}^{2}+V_{A}+V_{S}$$

$${\text{Inaccuracy}}_{\mathrm{Low}}={\left({\bar{a}}_{\text{fire}}-{\bar{s}}_{\text{fire}}\right)}^{2}+V_{a}+V_{s}$$

Inaccuracy_no‐fire S ⇄ fire L_:

$${\text{Inaccuracy}}_{\text{High}}={\left({\bar{A}}_{\mathrm{no}-\text{fire}}-{\bar{S}}_{\text{fire}}\right)}^{2}+V_{A}+V_{S}$$

$${\text{Inaccuracy}}_{\mathrm{Low}}={\left({\bar{a}}_{\text{fire}}-{\bar{s}}_{\mathrm{no}-\text{fire}}\right)}^{2}+V_{a}+V_{s}$$

Inaccuracy_no‐fire L ⇄ fire S_:

$${\text{Inaccuracy}}_{\text{High}}={\left({\bar{A}}_{\text{fire}}-{\bar{S}}_{\mathrm{no}-\text{fire}}\right)}^{2}+V_{A}+V_{S}$$

$${\text{Inaccuracy}}_{\mathrm{Low}}={\left({\bar{a}}_{\mathrm{no}-\text{fire}}-{\bar{s}}_{\text{fire}}\right)}^{2}+V_{a}+V_{s}$$

where A = high anther, a = low anther, S = high stigma, s = low stigma (Fig. 1A), and V = variance.

The first part of the equations (in bold) takes the squared difference between the organ means and refers to the maladaptive bias (i.e., a departure from optimum reciprocity), which we also calculated separately. The second part of the formula is composed of the organ variances, which refer to the imprecision of organs. Finally, we calculated the total inaccuracy as the sum of high and low inaccuracies (Armbruster *et al*. 2017; Trevizan *et al*. 2021a).

### Floral reward and pollen deposition

We collected accumulated nectar to quantify nectar availability and quality. Thus, we sampled ca. 10 flowers per morph per area (Table S1). Inflorescences with flowers in the pre‐anthesis stage were bagged on the previous day to avoid the assessment of floral visitors. Nectar was extracted at 12:00 h, and volume was estimated using capillary tubes, while sugar concentration was measured using a hand refractometer. Concentration measurements were converted into sucrose equivalents in μg sucrose per μL nectar (*sensu* Kearns & Inouye 1993). Then, we calculated nectar calories, assuming that every 1 mg of sugar corresponds to four calories (Dafni 1992).

We assessed pollen deposition on 382 flowers from 10 individuals and ca. 96 flowers per morph per area (Table S1). The flowers were collected on the next morning after being exposed to visitors for 1 day. The collected pistils were prepared on a slide, and the number of pollen grains deposited on stigmas was then counted under a microscope (Olympus BX51).

### Statistical analyses

To investigate floral trait differences, we compared their distribution in multivariate space, we first ran a principal components analysis (PCA) using the R‐package *FactoMineR* v. 2.4 (Lê *et al*. 2008) to reduce dimensionality and evaluate associations between measurements. We specified a covariance matrix since our variables were on the same scale (mm) (Abdi & Williams 2013). To visualize differences, we used the combination of morph per area as a supplementary variable for plotting. Next, we tested if the morphometric variables differed in the Euclidean space according to morph, area, and the interaction term by running a db‐RDA (distance‐based redundancy analysis; McArdle & Anderson 2001) with 10,000 permutations using the R‐package *vegan* v. 2.6.2 (Oksanen *et al*. 2022). We used the R‐package *usdm* v. 1.1.18 (Naimi *et al*. 2014) to investigate the variable's variance inflation factors (VIFs). We assumed that multicollinearity was not a problem because all values were ≤7.25 (below 10.0, as suggested by Borcard *et al*. 2018). After finding a significant effect of the interaction term, we conducted Bonferroni‐corrected pairwise analyses using the R‐package *pairwiseAdonis* v. 0.4 (Martinez Arbizu 2017).

Next, we used generalized linear models (GLMs) or generalized linear mixed models (GLMMs), when appropriate, to investigate the univariate effects of the several variables gathered according to morph (two levels: L‐ and S‐styled), area (two levels: no fire and fire), and the interaction term predictors (models 1–16; Table 1). We adjusted plant individual identity as a random effect in all models, except those testing the effects of individual traits, which consist of independent samplings. We used a Gaussian distribution with identity link for the models with the following response variables: corolla diameter, stigma height, nectar volume, nectar calories, and inflorescence length, and the gamma distribution (log‐link) for models with the response variables: plant height, stem diameter, corolla length, anther height, stigma length, and anther length. Count data were analysed using a negative binomial type 1 distribution (linear parameterization) for the variables: number of inflorescences, number of buds, number of fruits, and number of pollen grains deposited. We observed a significant excess of zeros for this latter response variable and then added a zero‐inflation parameter into the model. Since nectar concentration and pollen deposition were proportional data derived from continuous and count data, these were analysed by adjusting *beta* and *binomial* distributions (with logit link), respectively (Douma & Weedon 2019). We added a dispersion parameter to some models (i.e., *dispformula* function in *glmmTMB*) to account for heteroscedasticity and improve fit (Table 1). Whenever we found a significant effect for the interaction term, we used the R‐package *emmeans* v. 1.8.4.1 (Lenth *et al*. 2023) to conduct *post‐hoc* analysis using contrasts conditioning on morph to compare area type directly.

**Table 1:** Detailing of models, including the model parameters, the respective results, and graphs.

model	model parameters				results						figure
	response variable	family	link	additional parameter	area		morph		area * morph		
					χ^2^	*P*	*χ* ^2^	*P*	*χ* ^2^	*P*	
M.1	Plant height	Gamma	Log	Absent	3.42	0.064	2.20	0.137	0.99	0.318	Fig. S2A–C
M.2	Stem diameter	Gamma	Log	Absent	2.26	0.132	0.76	0.380	0.01	0.921	Fig. S2D–F
M.3	Number of inflorescences	Neg. bin. 1	Log	Absent	0.74	0.389	0.01	0.893	0.00	0.945	Fig. S2G–I
M.4	Inflorescence length	Gaussian	Identity	Absent	7.31	**0.007**	1.92	0.166	0.85	0.357	Fig. 2A–C
M.5	Number of buds	Neg. bin. 1	Log	Absent	0.05	0.816	0.13	0.715	1.92	0.166	Fig. S2J–L
M.6	Number of fruits	Neg. bin. 1	Log	Dispersion	2.70	0.101	2.37	0.124	9.17	**0.002**	Fig. 2D–F
M.7	Corolla length	Gamma	Log	Dispersion	8.11	**0.004**	0.68	0.409	3.45	0.063	Fig. 2G–I
M.8	Corolla diameter	Gaussian	Identity	Absent	0.68	0.410	0.36	0.550	0.11	0.735	Fig. S3A–C
M.9	Stigma height	Gaussian	Identity	Absent	0.75	0.387	209.14	**<0.001**	0.02	0.890	Fig. 3A–C
M.10	Anther height	Gamma	Log	Dispersion	0.02	0.879	220.15	**<0.001**	1.04	0.307	Fig. 3D–F
M.11	Stigma length	Gamma	Log	Absent	30.24	**<0.001**	150.35	**<0.001**	16.72	**<0.001**	Fig. 3G–I
M.12	Anther length	Gamma	Log	Dispersion	6.61	**0.010**	0.20	0.657	2.78	0.095	Fig. 3J–L
M.13	Nectar volume	Gaussian	Identity	Dispersion	0.01	0.933	2.38	0.123	2.85	0.091	Fig. S3D–F
M.14	Nectar concentration	Beta	Logit	Absent	0.16	0.690	0.77	0.380	0.08	0.766	Fig. S3G–I
M.15	Nectar calories	Gaussian	Identity	Absent	0.15	0.694	5.22	**0.022**	2.59	0.108	Fig. 2J–L
M.16	N° pollen grains deposited	Neg. bin. 1	Log	Zero‐infl.	2.66	0.10	0.99	0.32	1.18	0.28	Fig. S3J–L

Significant *P*‐values are in bold. Degrees of freedom = 1 in all cases. Symbol (*) indicates interaction term.

Neg. bin. 1, negative binomial distribution; Zero‐infl., Zero‐inflation.

We used the *glmmTMB* R‐package to fit all models (Brooks *et al*. 2017) and assessed their fit by inspecting the residual distribution after simulating these 250 times using the R‐package *DHARMa* v. 0.4.6 (Hartig & Lohse 2022). This latter package was also used to test for zero inflation. We used null statistical hypothesis significance testing using type II Wald chi‐square tests in the R‐package *car* v. 3.1.1 (Fox & Weisberg 2019). We report and plotted model‐adjusted (predicted) values, which were back‐transformed using the *ggeffect* function available in the R‐package *ggeffects* v. 1.1.5 (Lüdecke 2018). Analyses were performed in R software v. 4.2.2 (R Core Team 2024).

## RESULTS

The first (83.6%) and second (12.1%) PC axes on flower morphology explained together 95.7% of variation in the data (Fig. S1A,B). Stigma and anther heights contributed most to the first PC axis (Fig. S1C). These same two variables and corolla length were contributed most to the second PC axis (Fig. S1D). Based on these variables, we found that the largest separation in Euclidean space was due to the morph factor (db‐RDA—pseudo‐F_1,317_ = 1410.82, *P* < 0.001, *R* ^2^ = 0.80; Fig. S1A). Although explaining less variance, area (burned or unburned) and the interaction term were also significant (area: pseudo‐F_1,317_ = 16.80, *P* < 0.001, *R* ^2^ = 0.01; area × morph: pseudo‐F_1,317_ = 8.06, *P* = 0.003; *R* ^2^ = 0.005; Fig. S1A). Considering this latter, we found that all four groups differed from each other (all *P* <0.05).

**Fig. 2:**
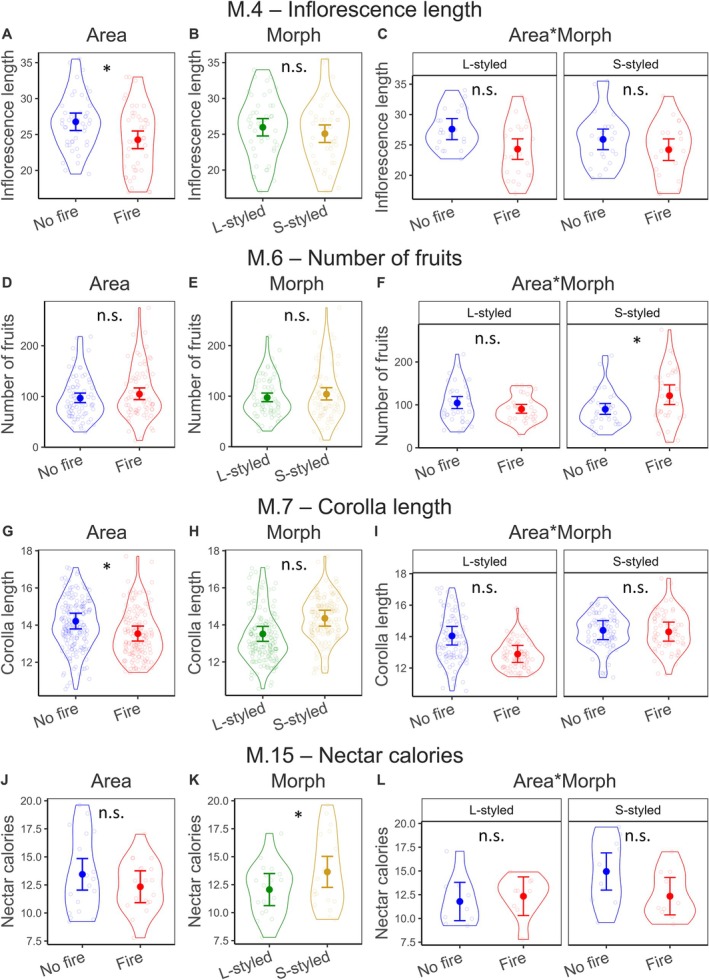
Results from the models testing the effects of area, morph, and their interaction term on the response variables: inflorescence length (M.4, A–C), number of fruits (M.6, D–F), corolla length (M.7, G–I), and nectar calories (J–L, M.15). Dots and line segments indicate back‐transformed predicted probabilities and 95% CIs, respectively. Empty points are the observed data, and violins show their respective distribution. Symbol (*) and n.s. indicate significant and non‐significant results, respectively.

Regarding the effects on individual traits, we did not find significant effects for area, morph, or the interaction term in determining plant height, stem diameter, number of inflorescences per individual, or the number of buds (M.1–M.4; Table 1, Fig. S2A–L). On the other hand, inflorescence length differed between areas, being 9.38% smaller in the area with fire when compared to the area without fire (marginal mean ± SE: 24.26 ± 0.62 vs. 26.77 ± 0.61, respectively; M.4; Table 1, Figs. 2A, 4). Morph or the interaction term did not influence inflorescence length (Fig. 2B,C). Considering number of fruits, there was a significant effect of the interaction term (M.6; Table 1). Post‐hoc analyses revealed that the effect was morph‐dependent, with only inflorescences of the S‐morph from the area with fire producing more fruits (35.31% increase) than those in the area without fire (121.47 ± 0.09 vs. 89.77 ± 0.07, respectively; Figs. 2F, 4). Neither area nor morph influenced the number of fruits (Fig. 2D,E).

**Fig. 3:**
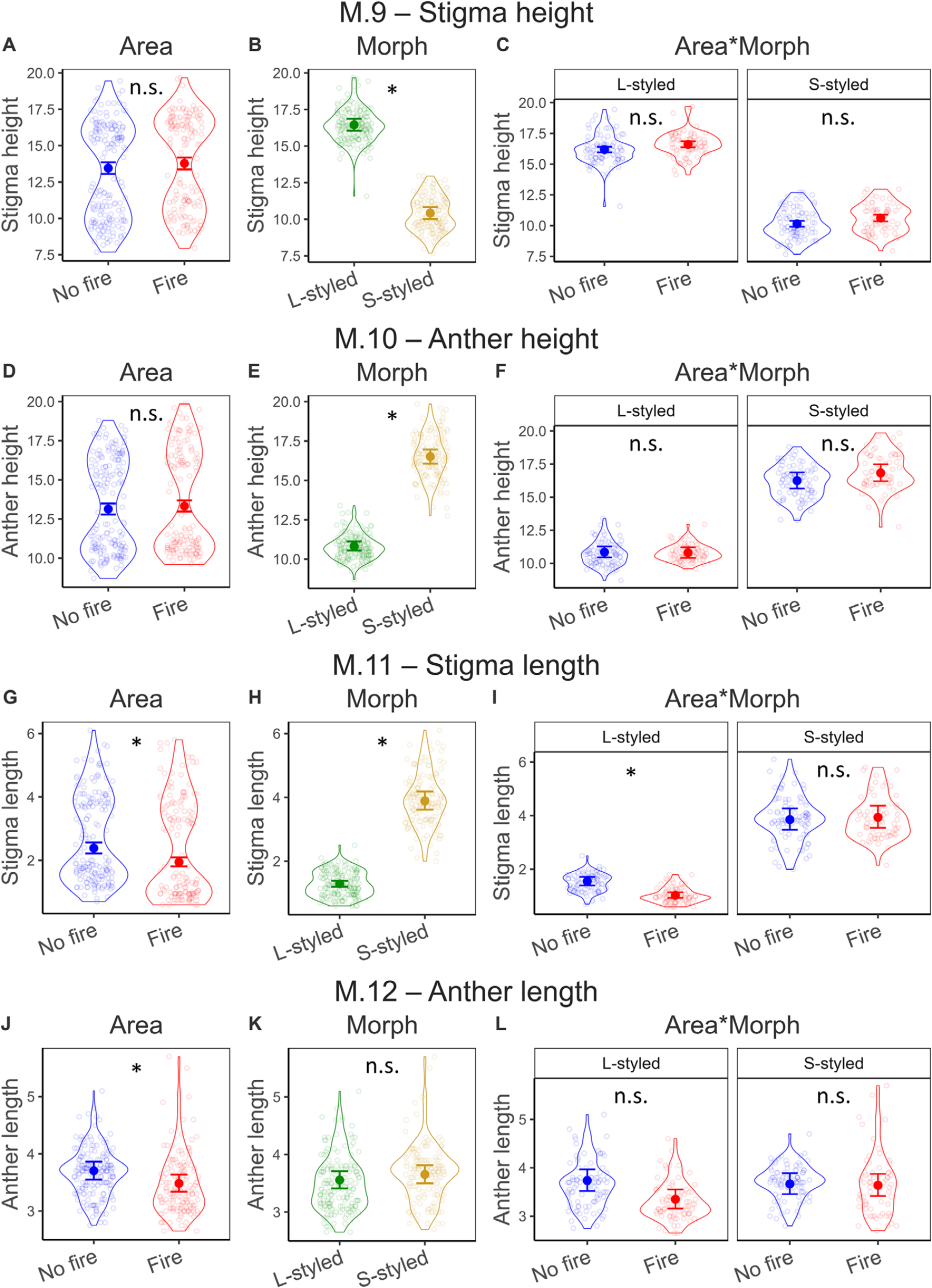
Results from the models testing the effects of area, morph, and their interaction term on the response variables: stigma height (A–C, M.9), anther height (D–F, M.10), stigma length (G–I, M.11), and anther length (J–L, M.12). Dots and line segments indicate back‐transformed predicted probabilities and 95% CIs, respectively. Empty points are the observed data, and violins show their respective distribution. Symbol (*) and n.s. indicate significant and non‐significant results, respectively.

**Fig. 4:**
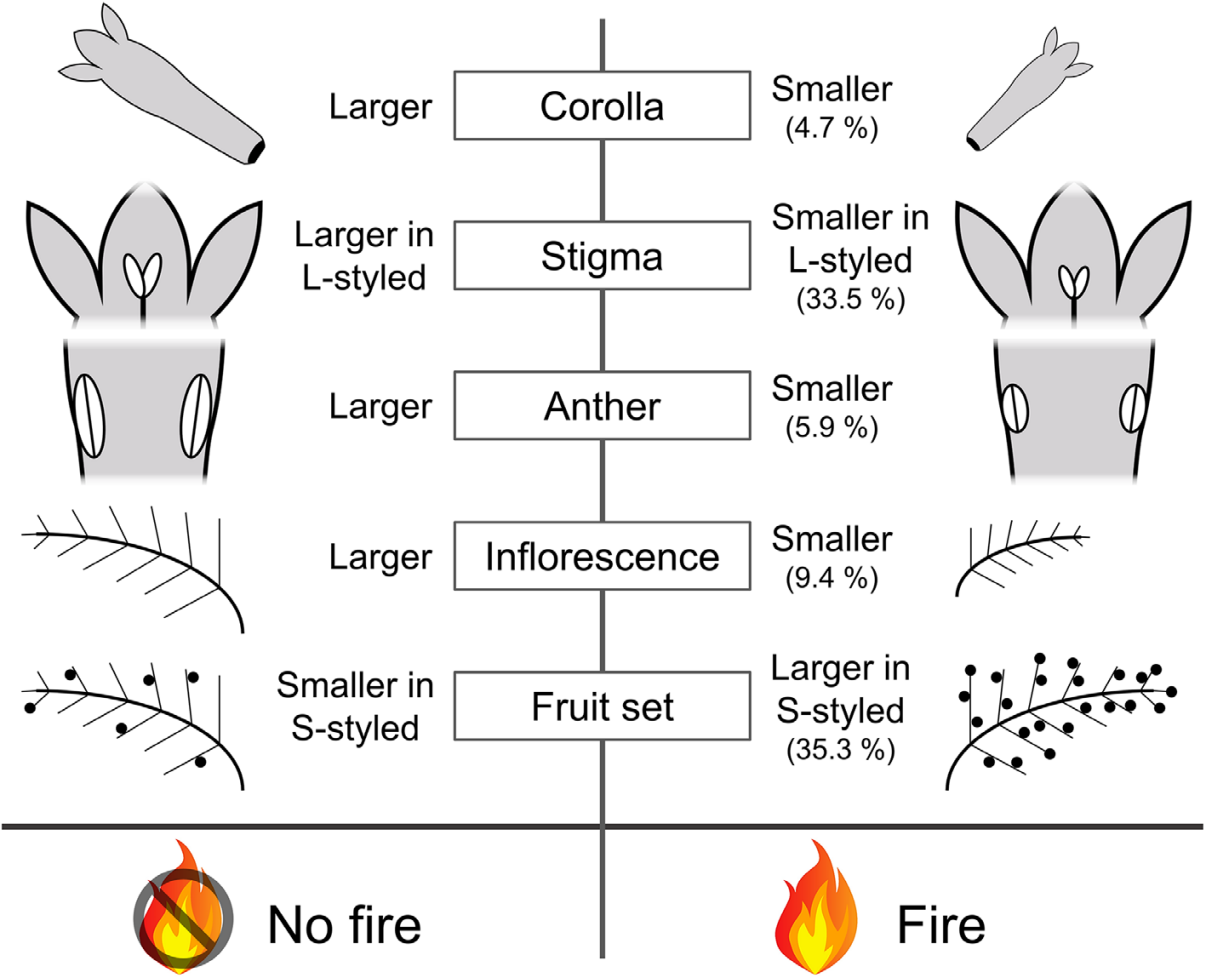
Schematic representation of our main findings showing the effects of fire on the morphological traits of *Palicourea rigida*. Comparisons show the effect sizes calculated based on the percentage of differences between predicted probabilities, with fire always being compared to no fire.

For the univariate effects concerning floral traits, we found that the model assessing differences in corolla length had a significant effect of area (M.7; Table 1). Corollas were 4.71% smaller in the area with fire compared with the area without fire (13.54 ± 0.02 vs. 14.21 ± 0.02, respectively; Figs. 2G, 4). Morph and the interaction term did not have any significant effects (Table 1 and Fig. 2H,I). None of the predictor variables significantly influenced corolla diameter (M.8; Table 1 and Fig. S3A–C). Considering stigma height, there was an effect of morph, with L‐styled stigmas being 57.97% taller than S‐styled stigmas (16.46 ± 0.21 vs. 10.42 ± 0.21, respectively; M.9; Table 1 and Fig. 3B). We did not find significant effects of area or the interaction term in stigma height (Table 1 and Fig. 3A,C). Anther height differed according to morph, with S‐styled being 52.45% higher than L‐styled (16.51 ± 0.01 vs. 10.83 ± 0.01, respectively; M.10; Table 1 and Fig. 3E). There were no significant effects of area or the interaction term on anther height (Table 1 and Fig. 3D,F). Considering stigma length, there were significant effects of area and the interaction term (M.11; Table 1 and Fig. 3G,I). Post‐hoc analyses indicated that the differences were restricted to the L‐styled morph, in which stigmas from the area with fire were 33.55% smaller than those of the area without fire (1.03 ± 0.05 vs. 1.55 ± 0.05, respectively; Figs. 3I, 4). Morph also influenced stigma length, with S‐styled morphs being 201.55% larger than L‐styled ones (3.89 ± 0.04 vs. 1.29 ± 0.04, respectively; Fig. 3H). Concerning anther length, there was an effect of area, with anthers being 5.95% smaller in the area with fire when compared to the unburned area (3.48 ± 0.02 vs. 3.70 ± 0.02, respectively; M.12; Table 1 and Figs. 3J, 4). Morph and the interaction term did not have any significant effects on anther size (Fig. 3K,L).

There were similarities among the inaccuracy values and combinations. Although all four combinations followed the same pattern without major differences, in general, the fire L ⇄ fire S combination had the lowest values of inaccuracy (Fig. 1B and Table S2), while the no fire ⇄ fire combination had slightly higher values (Fig. 1B and Table S2). The other combinations had intermediate values (Fig. 1B and Table S2). While values of maladaptive bias were low for all combinations, the increment in the inaccuracies of both high and low organs and total inaccuracy was, therefore, primarily due to the contribution of organ variances.

Considering nectar traits, area, morph, or interaction term did not influence nectar volume or nectar concentration (M.13 and M.14; Table 1 and Fig. S3D–I). Considering nectar calories, morph had a significant effect, with S‐styled morphs presenting 13.10% more calories than L‐styled morphs (13.64 ± 0.68 vs. 12.06 ± 0.71, respectively; M.15; Table 1 and Fig. 2K). However, neither the area or the interaction term affected the amount of nectar calories (Fig. 2J,L). Concerning the number of pollen grains deposited on stigmas, we did not find effects of area, morph, or the interaction term (M.16; Table 1 and Fig. S3J–L).

## DISCUSSION

This study demonstrates, for the first time, the effects of fire on pollination‐dependent floral polymorphisms. Among the various individual and floral traits, fire affected the length of some structures, resulting in smaller inflorescences, corolla, anthers, and L‐styled stigmas, which can be attributed to post‐resprouting developmental limitations. However, fire did not affect attractiveness and reward traits, including plant height, number of inflorescences, number of floral buds, and nectar attributes. Importantly, the fire did not influence height of the stigmas and anthers, and reciprocity, which are the most fundamental aspects of the pollination process in distylous plants. This resilience enabled recently burned plants to receive legitimate pollen grains, resulting in fruit formation, which was even higher in S‐styled individuals. Despite the punctual negative effects of fire, we confirm our research hypothesis that organ height remains unchanged, translating into distyly functioning and plant reproduction. These results show that recently burned plants can quickly resprout and maintain pollen flow within fire‐affected areas and across the vegetation mosaic, including unburned patches.

We also found that fire does not influence most individual traits, including plant height, stem diameter, number of buds, and number of inflorescences. Plant height and number of inflorescences are important display traits for pollinator attraction (Klinkhamer *et al*. 1989; Klinkhamer & Jong 1990; Ohashi 2001; Brys *et al*. 2008), indicating that fire‐affected plants maintain similar attractivity to those without fire experience. Many plant species in savanna ecosystems rapidly recover through a resprouting strategy (Hoffmann *et al*. 2009; Simon *et al*. 2009; Pilon *et al*. 2021). Fire also did not influence resource offering, including nectar volume, concentration, and calories, indicating that burned plants can reward pollinators in similar ways. Even considering the idiosyncrasy of fire intensity and the fact that our data refer to a small time scale (one season), our results confirm that *P. rigida* is highly resilient to fire, with the aerial part of individuals surviving and still showing rapid crown resprouting. This allows for the successful flowering of burned plants and nectar production at the appropriate time. It also demonstrates how burned plants remain synchronized with the overall phenology of the population, which may allow pollen flow between burned and unburned plants in the mosaic of burned and unburned patches created by fire in the landscape. The rapid post‐fire flowering involves complex physiological responses (Ferraro *et al*. 2021; Santacruz‐García *et al*. 2021; Rosalem *et al*. 2022; Ferreira *et al*. 2023) and may be part of many adaptations to fire shown by Cerrado plants (Simon *et al*. 2009).

Fire reduced some morphological traits, such as inflorescence length, corolla, and anthers. It also had morph‐specific effects in the case of L‐styled stigmas, indicating how distinct morphs can respond differentially to this disturbance. After the fire, plants usually display larger floral characters linked to increased provisioning of reserves and nutrients released by the ashes of the burned biomass (Lamont & Downes 2011; Clarke *et al*. 2013; Fidelis & Zirondi 2021; Zirondi *et al*. 2021; Salim *et al*. 2022). On the other hand, smaller traits, as observed here, may be a response to a costly resprouting process that requires complex post‐fire physiological responses (Clarke *et al*. 2013; Santacruz‐García *et al*. 2021; Rosalem *et al*. 2022). Our field data were gathered 2 months after the fire, when considerable energy had already been redirected towards resprouting, which allowed the plants to flower at the usual flowering time and reproduce within the same year. Thus, the smaller floral traits observed are probably explained by an energy trade‐off between the post‐fire developmental limitations of regrowth and the size of the structures.

The smaller morphological floral traits could affect pollination success between the fire and non‐fire areas. Slight differences in floral morphology can affect pollination efficiency of distylous flowers (Ganders 1979). For instance, the corolla length variation can reduce herkogamy and decrease compatible pollen deposition in distylous plants (Liu *et al*. 2016). In addition, lower corollas can potentially affect the height of S‐styled anthers because *P. rigida* has epipetalous stamens, a common trait in the Rubiaceae (Trevizan *et al*. 2021a). Thus, we suggest that the differences in corolla length found here could have caused a mismatch between anthers and stigma heights, therefore influencing the efficiency of pollen transfer between morphs in the mosaic of burned and unburned areas. However, fire did not affect the effective height of reproductive organs, which are the most important trait in the distyly system, ensuring precise pollination (Keller *et al*. 2014). Differences in organ heights were only restricted to within‐morph traditional morphologies, corroborating the typical distylous characteristics of this species (Machado *et al*. 2010; Trevizan *et al*. 2021a; Cardoso *et al*. 2022). For instance, whorl differences in multivariate space were explained mainly by morph (80%), with the area factor having a minor effect (1%). Thus, even though the fire has immediate negative effects on some traits, organ height positions were consistent, maintaining adequate deposition on pollinator bodies and guaranteeing sexual reproduction. In agreement, such similarity in organ heights leads to similar inaccuracies between morphs within areas and among fire and no‐fire area combinations, suggesting consistent reciprocity between morphs. Together, these results support a conservative arrangement in reproductive traits, ensuring the dispersal of legitimate intermorph pollen grains across both the fire and no‐fire areas.

Our data on reproductive output suggest that such a fire event does not affect the sexual reproduction of this distylous population. The number of pollen grains deposited onto stigmas was similar across morphs and areas, indicating that pollen dispersal occurred effectively in all plants in the population. Pollinators in fire‐prone environments may show distinct responses, with birds showing a tendency for negative effects, although with significant variation (Carbone *et al*. 2019). In Cerrado, low‐frequency fires create a mosaic of burned and unburned areas in the landscape that do not strongly affect plant–pollinator interactions (Baronio *et al*. 2021). In agreement, the fire was not detrimental to fruit formation. On the contrary, it had a morph‐dependent effect, with S‐styled morphs showing higher fruit set rates after fire. *Palicourea rigida* has only two ovules per fruit, so a few pollen grains can be sufficient for successful pollination (Machado *et al*. 2010). Since many pollen grains reach the stigmas, higher fruit set in the fire area can be attributed to a physiological control, allowing greater permissibility for ovule penetration and/or reduced abortion rate. Thus, the higher fruit formation may be related to the increased availability of nutrients that promote plant vigour and lead to higher ovule fecundity and fruit production (Franceschinelli & Bawa 2005; Carbone *et al*. 2025).

While fire has been present in the Cerrado for at least four million years (Simon *et al*. 2009), *P. rigida* is one of the most widely distributed plants in the biome (Bridgewater *et al*. 2004). The high resprouting capacity, associated with rapid restoration of reproductive capacity of a system sensitive to subtle variations and strictly dependent on pollinators, may help explain this plant's success and shed light on the resilience that floral traits can show in relation to this ancient abiotic factor. However, we acknowledge that our study is based on a single population of a fire‐adapted species and after a single fire episode. Given the idiosyncratic and often complex responses of plants to fire, further studies should investigate other *P. rigida* populations with different fire histories and adopt long‐term approaches, in addition to investigating other plant species with different pollination and growth strategies.

## CONCLUSION

We show that the flowering, floral traits, and sexual reproduction of *P. rigida* are fire‐adapted, with the species having a high capacity for recovery through a resprouting strategy, which favours its re‐establishment soon after the fire, leading to the simultaneous flowering of plants in both fire and non‐fire areas and facilitating pollen transfer. Even though we found some negative effects of fire, including inflorescence, corolla, anther, and L‐morph stigmas length, we found that fire did not affect plant height, number of inflorescences, resource availability, and especially stigma–anthers heights, promoting reciprocity between morphs within fire‐affected areas and between burned and unburned plants in the population. This ensures intermorph pollen transfer and maintains the functioning of the floral polymorphism. Hence, our findings suggest that plants are highly resilient in the face of fire events, and maintain a conserved distyly system that ensures suitable morphological traits, pollen flow, and deposition between morphs, securing sexual reproduction of the plants affected by fire. To the best of our knowledge, our study is the first to explore the effects of fire on traits in floral polymorphisms. It provides valuable insights into the adaptations and flexibility of floral polymorphisms, which have endured for thousands of years in fire‐prone ecosystems. Such plant resilience may prove even more important for maintaining polymorphic plant diversity and the associated fauna of pollinators when considering the increasingly frequent anthropogenic fires.

## AUTHOR CONTRIBUTIONS

The design of the study, material preparation, and data collection were done by CM, FWA, and PKM. Analysis, figures, and writing of the first manuscript draft were done by RT and JCFC. All authors read, provided suggestions, and approved the final version of the manuscript.

## FUNDING INFORMATION

This study is part of R.T.'s doctoral thesis (Souza 2024). R.T. and J.C.F.C. are grateful to Coordenação de Aperfeiçoamento do Pessoal de Nível Superior (CAPES)—Finance code 001 for a doctoral fellowship at the University of Campinas (UNICAMP) and a postdoctoral grant at the Universidade Federal de Minas Gerais (UFMG; project n°88887.006179/2024‐00), respectively. R.T. is grateful to Fundação de Amparo à Pesquisa do Estado de Minas Gerais (FAPEMIG) for her postdoctoral grant (RED‐00039‐23). F.W.A. thanks Conselho Nacional de Desenvolvimento Científico e Tecnológico—CNPq (grant #308559/2022‐3). We are also grateful to Fundação de Amparo à Pesquisa do Estado de Minas Gerais (FAPEMIG; RED‐00039‐23 and APQ‐03249‐22).

## Supporting information


**Fig. S1.** Principal component analysis (PCA) results. (A) Biplot showing the contributions and correlations of morphological traits according to area and morph. Points show observed data, and ellipses comprise 0.95 CI. (B) Scree plot showing the percentage of explained variances of each PC. (C, D) Percentage of contributions of variables to PC1 and PC2. The red dashed reference line indicates the expected value if all the contributions were uniform.
**Fig. S2.** Results from the models testing the effects of area, morph, and their interaction term on the variables: plant height (A–C, M.1), stem diameter (D–F, M.2), number of inflorescences (G–I, M.3), and number of buds (J–L, M.5) of *Palicourea rigida*. Dots and line segments indicate back‐transformed predicted probabilities and 95 % CIs, respectively. Empty points are the observed data, and violins show their respective distribution. n.s. indicate non‐significant results.
**Fig. S3.** Results from the models testing the effects of area, morph, and their interaction term on the variables: corolla diameter (A–C, M.8), nectar volume (D–F, M.13), nectar concentration (G–I, M.14), and number of pollen grains deposited (J‐L, M. 16) of *Palicourea rigida*. Dots and line segments indicate back‐transformed predicted probabilities and 95 % CIs, respectively. Empty points are the observed data, and violins show their respective distribution. n.s. indicate non‐significant results.

**Table S1.** Number of individuals sampled and sample sizes of the response variables used in models. Model specifications can be found in Table 1.
**Table S2.** Inaccuracy values of legitimate combinations of reproductive structures of distylous *Palicourea rigida*. These results are shown in Figure 1B.
