# Supplementary material for: Investigating the effects of fire on pollinator‐dependent distyly polymorphism
Source: Plant Biol (Stuttg). 2025 Jul 4;27(6):1035–46. doi: 10.1111/plb.70062 (PMC12477302; doi:10.1111/plb.70062)
Supplement: Supplementary file 1 — Fig. S1. Principal component analysis (PCA) results. (A) Biplot showing the contributions and correlations of morphological traits according to area and morph. Points show observed data, and ellipses comprise 0.95 CI. (B) Scree plot showing the percentage of explained variances of each PC. (C, D) Percentage of contributions of variables to PC1 and PC2. The red dashed reference line indicates the expected value if all the contributions were uniform. Fig. S2. Results from the models testing the effects of area, morph, and their interaction term on the variables: plant height (A–C, M.1), stem diameter (D–F, M.2), number of inflorescences (G–I, M.3), and number of buds (J–L, M.5) of Palicourea rigida. Dots and line segments indicate back‐transformed predicted probabilities and 95 % CIs, respectively. Empty points are the observed data, and violins show their respective distribution. n.s. indicate non‐significant results. Fig. S3. Results from the models testing the effects of area, morph, and their interaction term on the variables: corolla diameter (A–C, M.8), nectar volume (D–F, M.13), nectar concentration (G–I, M.14), and number of pollen grains deposited (J‐L, M. 16) of Palicourea rigida. Dots and line segments indicate back‐transformed predicted probabilities and 95 % CIs, respectively. Empty points are the observed data, and violins show their respective distribution. n.s. indicate non‐significant results. [file PLB-27-1035-s001.zip › Figures S1-3 Caption.docx]

**Figure S1:** Principal component analysis (PCA) results. (A) Biplot showing the contributions and correlations of morphological traits according to area and morph. Points show observed data, and ellipses comprise 0.95 CI. (B) Scree plot showing the percentage of explained variances of each PC. (C, D) Percentage of contributions of variables to PC1 and PC2. The red dashed reference line indicates the expected value if all the contributions were uniform.

**Figure S2:** Results from the models testing the effects of area, morph, and their interaction term on the variables: plant height (A–C, M.1), stem diameter (D–F, M.2), number of inflorescences (G–I, M.3), and number of buds (J–L, M.5) of *Palicourea rigida*. Dots and line segments indicate back-transformed predicted probabilities and 95 % CIs, respectively. Empty points are the observed data, and violins show their respective distribution. n.s. indicate significant and non-significant results, respectively.

**Figure S3:** Results from the models testing the effects of area, morph, and their interaction term on the variables: corolla diameter (A–C, M.8), nectar volume (D–F, M.13), nectar concentration (G–I, M.14), and number of pollen grains deposited (J-L, M. 16) of *Palicourea rigida*. Dots and line segments indicate back-transformed predicted probabilities and 95 % CIs, respectively. Empty points are the observed data, and violins show their respective distribution. n.s. indicate significant and non-significant results, respectively.
