# Supplementary material for: Investigating the effects of fire on pollinator‐dependent distyly polymorphism
Source: Plant Biol (Stuttg). 2025 Jul 4;27(6):1035–46. doi: 10.1111/plb.70062 (PMC12477302; doi:10.1111/plb.70062)
Supplement: Supplementary file 2 — Table S1. Number of individuals sampled and sample sizes of the response variables used in models. Model specifications can be found in Table 1. Table S2. Inaccuracy values of legitimate combinations of reproductive structures of distylous Palicourea rigida. These results are shown in Figure 1B. [file PLB-27-1035-s002.docx]

| Model | Response variable | Number of individuals | | | | | Number of replicates (flowers/inflorescences/individuals) | | | | |
| --- | --- | --- | --- | --- | --- | --- | --- | --- | --- | --- | --- |
|  |  | No fire | | Fire | | Total | No fire | | Fire | | Total |
|  |  | L-styled | S-styled | L-styled | S-styled |  | L-styled | S-styled | L-styled | S-styled |  |
| M.1 | Plant height | 13 | 19 | 21 | 16 | 69 | 13 | 19 | 21 | 16 | 69 |
| M.2 | Stem diameter | 13 | 19 | 21 | 16 | 69 | 13 | 19 | 21 | 16 | 69 |
| M.3 | Number of inflorescences | 13 | 19 | 21 | 16 | 69 | 13 | 19 | 21 | 16 | 69 |
| M.4 | Inflorescence length | 22 | 23 | 23 | 21 | 89 | 22 | 23 | 23 | 21 | 89 |
| M.5 | Number of buds | 22 | 23 | 23 | 21 | 89 | 22 | 23 | 23 | 21 | 89 |
| M.6 | Number of fruits | 12 | 12 | 10 | 10 | 44 | 38 | 35 | 33 | 36 | 142 |
| M.7 | Corolla length | 10 | 10 | 10 | 10 | 40 | 90 | 86 | 79 | 66 | 321 |
| M.8 | Corolla diameter | 10 | 10 | 10 | 10 | 40 | 90 | 86 | 79 | 66 | 321 |
| M.9 | Stigma height | 10 | 10 | 10 | 10 | 40 | 90 | 86 | 79 | 66 | 321 |
| M.10 | Anther height | 10 | 10 | 10 | 10 | 40 | 90 | 86 | 79 | 66 | 321 |
| M.11 | Stigma length | 10 | 10 | 10 | 10 | 40 | 90 | 86 | 79 | 66 | 321 |
| M.12 | Anther length | 10 | 10 | 10 | 10 | 40 | 90 | 86 | 79 | 66 | 321 |
| M.13 | Nectar volume | 3 | 3 | 3 | 3 | 12 | 9 | 10 | 9 | 10 | 38 |
| M.14 | Nectar concentration | 3 | 3 | 3 | 3 | 12 | 9 | 10 | 9 | 10 | 38 |
| M.15 | Nectar calories | 3 | 3 | 3 | 3 | 12 | 9 | 10 | 9 | 10 | 38 |
| M.16 | N° pollen grains deposited | 10 | 10 | 10 | 10 | 40 | 96 | 91 | 97 | 98 | 382 |

**SUPPORTING INFORMATION**

**Table S1**. Number of individuals sampled and sample sizes of the response variables used on models. Model specifications can be found in Table 1.

**Table S2.** Inaccuracy values of legitimate combinations of reproductive structures of distylous *Palicourea rigida*. These results are shown in Figure 1B.

| Inaccuracy values | Combinations | | | |
| --- | --- | --- | --- | --- |
|  | **no fire – no fire** | **fire - fire** | **no fire S – fire L** | **no fire L – fire S** |
| Maladaptive bias - High | 0.0005 | 0.127 | 0.207 | 0.619 |
| Maladaptive bias - Low | 0.316 | 0.025 | 0.396 | 0.008 |
| Inaccuracy High organs | 2.878 | 2.979 | 2.677 | 3.879 |
| Inaccuracy Low organs | 2.483 | 1.688 | 2.158 | 2.076 |
| Total | 5.362 | 4.668 | 4.835 | 5.956 |
